# Optimizing Inhaled Corticosteroid Prescribing in COPD: Evaluating the Impact of Eosinophil Testing on Clinical Outcomes

**DOI:** 10.1007/s00408-026-00930-x

**Published:** 2026-08-29

**Authors:** Jared Hester, David Whipkey, James Ferrell

**Affiliations:** 1 Department of Pharmacy, Cleveland Clinic Akron General, Akron, OH 44307 USA; 2 Department of Pharmacy Practice, Northeast Ohio Medical University, OH 44272 Rootstown, USA

**Keywords:** Chronic obstructive pulmonary disease, Inhaled corticosteroids, Eosinophils, Pneumonia, Guideline adherence, De-escalation

## Abstract

**Purpose:**

Inhaled corticosteroids (ICS) are recommended for select patients with chronic obstructive pulmonary disease (COPD) based on exacerbation history and blood eosinophil counts. Prior studies suggest that ICS are frequently prescribed outside of guideline-based criteria, and eosinophil testing is inconsistently performed in clinical practice. This study evaluated adherence to Global Initiative for Chronic Obstructive Lung Disease (GOLD) eosinophil criteria for ICS prescribing and associated clinical outcomes.

**Methods:**

This retrospective observational cohort study included adults with COPD receiving care within Cleveland Clinic outpatient practices in Ohio who were newly initiated on an ICS-containing regimen between January 1, 2021, and July 31, 2023. Patients were classified based on whether ICS prescribing aligned with GOLD eosinophil criteria. Outcomes included adherence to GOLD eosinophil recommendations for ICS use, pneumonia incidence, ICS de-escalation, and predictors of prescribing outside guideline-based criteria.

**Results:**

A total of 688 patients were included. Most patients received ICS outside of GOLD eosinophil criteria (589/688, 86%). Baseline eosinophil testing was performed in 102 patients (15%). Pneumonia incidence was similar among patients prescribed ICS outside versus within guideline-based criteria (15% vs. 16%, *p* = 0.686). ICS de-escalation was uncommon overall (7%) and occurred at similar rates between groups. A history of prior exacerbations was associated with prescribing within guideline-based indications.

**Conclusion:**

ICS prescribing frequently occurred outside of GOLD eosinophil criteria, with limited utilization of eosinophil testing in clinical practice. Clinical outcomes were similar regardless of guideline alignment, and ICS de-escalation was infrequent. These findings highlight opportunities to improve integration of eosinophil-guided decision-making into COPD management.

## Introduction

Chronic obstructive pulmonary disease (COPD) remains a leading cause of morbidity and mortality worldwide and is known to contribute substantially to healthcare utilization through exacerbations and progressive respiratory impairment. Pharmacologic management aims to reduce symptoms, improve quality of life, and prevent exacerbations using bronchodilator therapy and selective use of inhaled corticosteroids (ICS) [1]. Historically, ICS therapy was prescribed for patients with moderate-to-severe COPD with frequent exacerbations, which was also seen in patients with asthma. However, accumulating evidence has demonstrated that the benefits of ICS therapy vary significantly based on patients’ specific COPD phenotypes, leading to a more individualized approach to treatment. The current Global Initiative for Chronic Obstructive Lung Disease (GOLD) guidelines recommend ICS primarily for patients with frequent exacerbations and elevated blood eosinophil counts, as patients with low eosinophil counts are less likely to benefit and may be exposed to unnecessary risks [1, 2]. Despite these recommendations, observational studies suggest between 50 to 80% of patients with COPD receive ICS without meeting the guideline-based criteria, highlighting a persistent gap between evidence-based recommendations and real-world prescribing practices [3, 4].

Blood eosinophil counts have emerged as an important biomarker for predicting response to ICS therapy. Several studies have demonstrated that patients with higher eosinophil counts derive greater reductions in COPD exacerbations with ICS treatment, whereas patients with lower eosinophil counts experience minimal benefit [5, 6]. Post hoc analyses of large, randomized trials, including IMPACT and ETHOS, further demonstrated that the benefits of ICS-containing regimens increase with higher eosinophil counts, while patients with low eosinophil counts experience less clinical benefits [7, 8]. These findings contributed to the incorporation of blood eosinophil thresholds into GOLD recommendations beginning in 2019 [9]. Current guidelines recommend ICS therapy for patients with eosinophil counts ≥ 300 cells/µL because they are more likely to benefit from treatment and suggest consideration of ICS therapy in patients with eosinophil counts of 100–299 cells/µL when accompanied by a history of frequent exacerbations [1, 2, 9]. In contrast, patients with eosinophil counts < 100 cells/µL are unlikely to derive meaningful benefit and may be exposed to unnecessary risks associated with ICS therapy [1, 2, 9]. The FLAME trial demonstrated that dual bronchodilator therapy with LABA/LAMA was superior to LABA/ICS in reducing exacerbations and was associated with a lower risk of pneumonia, supporting a bronchodilator-first approach and more selective use of ICS therapy [10]. Additionally, Quint et al. reported that only approximately 10% of patients with COPD have eosinophil levels associated with a clear therapeutic benefit from ICS therapy, suggesting that a substantial proportion of patients may be exposed to ICS without a strong evidence-based indication [3].

Despite increasing emphasis on eosinophil-guided therapy, significant uncertainty remains regarding real-world adherence to these recommendations. Several studies have suggested that ICS therapy is frequently prescribed without documentation of eosinophil testing or exacerbation risk, raising concerns about potential overuse of ICS-containing medications in COPD patient populations. In addition to the limited benefits observed among patients with low eosinophil counts, ICS therapy is associated with several adverse effects, most notably oral candidiasis and an increased risk of pneumonia [10–12]. However, limited data exist evaluating whether clinical outcomes, including pneumonia risk, differ between patients prescribed ICS within and outside GOLD eosinophil criteria. Additionally, studies evaluating ICS withdrawal have demonstrated that many patients can safely discontinue ICS therapy without worsening outcomes. The SUNSET trial demonstrated that withdrawal of ICS in patients receiving triple therapy (ICS/LABA/LAMA) did not result in increased exacerbation risk among patients with low eosinophil counts, further supporting the role of eosinophil-guided ICS use [13–15].

Understanding real-world prescribing patterns is critical to ensuring that ICS use aligns with contemporary GOLD recommendations. Evaluating eosinophil testing practices, pneumonia outcomes, and patterns of ICS de-escalation may help identify opportunities to reduce unnecessary ICS exposure and improve adherence to guideline-directed COPD management. Therefore, the objective of this study was to evaluate adherence to GOLD eosinophil criteria for ICS prescribing among patients with COPD and to assess associated clinical outcomes, including pneumonia incidence and ICS de-escalation. These findings may help inform targeted provider education and quality improvement initiatives aimed at improving evidence-based COPD management and reducing unnecessary ICS exposure.

## Methods

### Study Design

This retrospective observational cohort study evaluated adult patients with COPD receiving care within Cleveland Clinic outpatient practices throughout Ohio. Patients newly initiated on an ICS-containing regimen between January 1, 2021, and July 31, 2023, were identified. Patients were followed for up to two years following ICS initiation or until death or loss to follow-up. This study was reviewed and approved by the Cleveland Clinic Institutional Review Board.

### Patient Selection

Adult patients aged 18 years or older with a diagnosis of COPD and classified as GOLD Group B or E who were newly initiated on an ICS-containing regimen during the study period were eligible for inclusion. Eligible regimens included ICS/long-acting beta agonist (LABA), ICS/long-acting muscarinic antagonist (LAMA), and ICS/LABA/LAMA combinations prescribed by outpatient providers within Cleveland Clinic Ohio.

Patients were excluded if they had a co-diagnosis of asthma, received ICS monotherapy, documented immunosuppression or use of immunosuppressive medications, lacked eosinophil testing during the study period, had incomplete medication or follow-up documentation, lacked an outpatient follow-up visit within six months of ICS initiation, or had a diagnosis of alpha-1 antitrypsin deficiency or cystic fibrosis. Patients were also excluded if eosinophil testing was obtained only during acute hospitalization or within 30 days of systemic corticosteroid exposure. Each patient was included once using the first qualifying ICS initiation during the study period.

### Patient Characteristics

Patient characteristics collected included age, sex, smoking status, comorbidities, COPD exacerbation history, prescriber specialty, prescriber credential, and ICS regimen type. Baseline eosinophil count was defined as the blood eosinophil value closest to and within three months prior to ICS initiation.

COPD exacerbations were categorized as moderate or severe. Moderate exacerbations were defined as those requiring treatment with systemic corticosteroids and/or antibiotics in the outpatient setting. Severe exacerbations were defined as exacerbations resulting in hospitalization. Prescriber specialty was categorized as pulmonology, internal medicine, family medicine, or other specialties.

### Outcomes

Baseline eosinophil testing availability was defined as the presence of a documented eosinophil count within three months prior to ICS initiation that was not obtained during an acute hospitalization and was not associated with systemic corticosteroid use within the preceding 30 days. The frequency of baseline eosinophil testing was reported for all included patients.

Patients were classified according to GOLD eosinophil criteria for ICS use. Guideline-concordant ICS prescribing was defined as a baseline eosinophil count ≥ 300 cells/µL or a baseline eosinophil count of 100–299 cells/µL in the presence of frequent exacerbations, defined as ≥ 1 severe exacerbation or ≥ 2 moderate exacerbations during the preceding year. Prescribing outside GOLD eosinophil criteria was defined as ICS initiation in patients with a baseline eosinophil count < 100 cells/µL or a baseline eosinophil count of 100–299 cells/µL without a qualifying exacerbation history.

The primary outcome was the proportion of patients prescribed ICS therapy outside GOLD eosinophil criteria. Secondary outcomes included the incidence of pneumonia, ICS de-escalation, time to ICS de-escalation, and predictors of prescribing outside guideline-recommended indications.

Pneumonia was identified using International Classification of Diseases, Tenth Revision (ICD-10) diagnosis codes or documentation within the electronic medical record supported by radiographic findings and/or antibiotic treatment. ICS de-escalation was defined as either reduction in ICS dose or discontinuation of an ICS-containing regimen during follow-up.

### Statistical Analysis

Descriptive statistics were used to summarize baseline characteristics and study outcomes. Continuous variables are reported as mean ± standard deviation (SD) or median with interquartile range (IQR), as appropriate. Categorical variables are reported as frequencies and percentages.

Comparisons between patients prescribed ICS within and outside GOLD eosinophil criteria were performed using Student’s t-test or the Mann–Whitney U test for continuous variables and the chi-square test or Fisher’s exact test for categorical variables, as appropriate. Odds ratios (ORs) with 95% confidence intervals (CIs) were calculated to identify predictors of prescribing outside guideline-recommended indications. A two-sided p-value < 0.05 was considered statistically significant. Statistical analyses were performed using Stata Statistical Software: Release 19 (StataCorp LLC, College Station, TX).

## Results

### Patient Characteristics

**Fig. 1:**
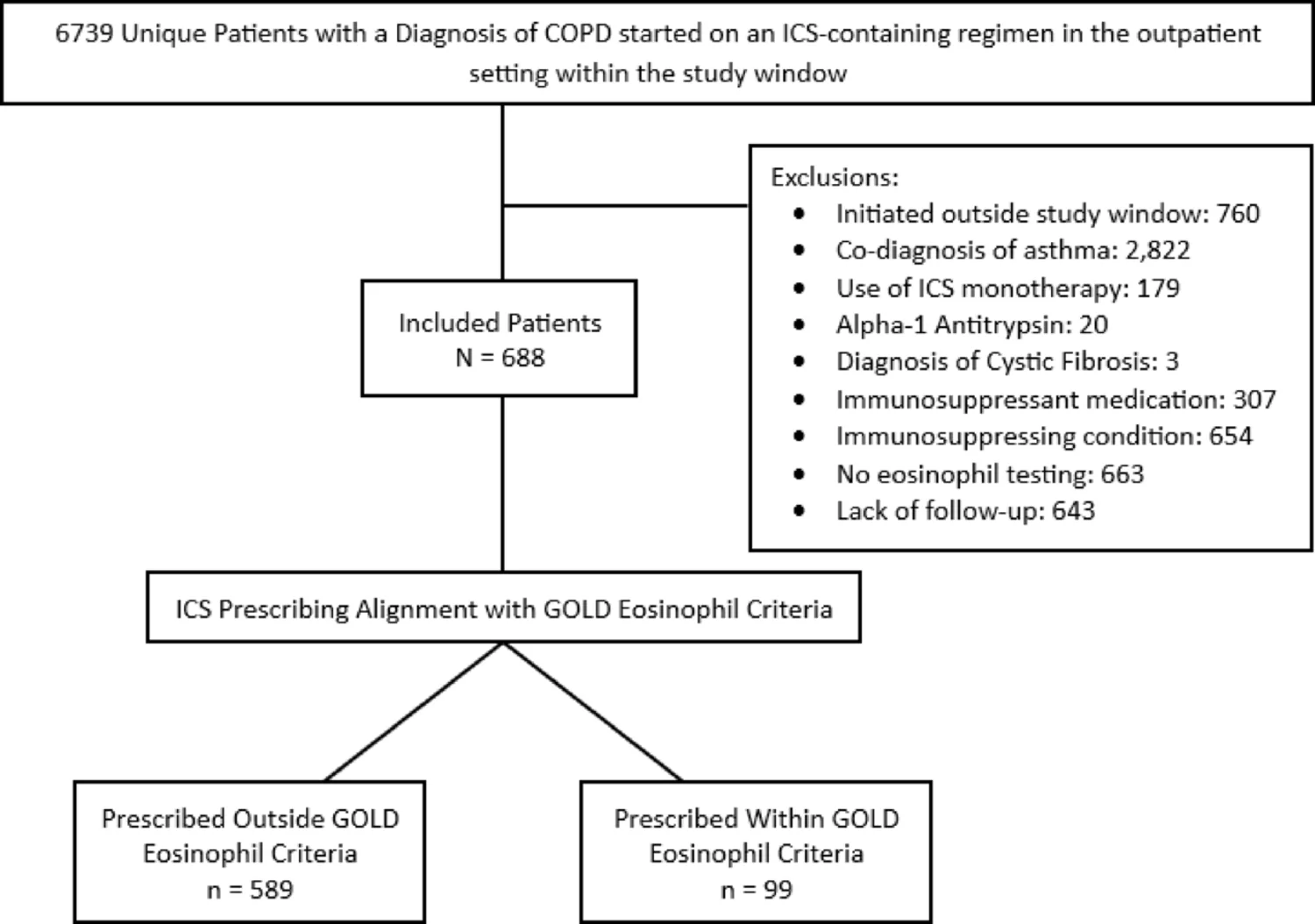
Study flow diagram depicting patient identification, exclusion criteria, and classification according to alignment of inhaled corticosteroid (ICS) prescribing with Global Initiative for Chronic Obstructive Lung Disease (GOLD) eosinophil criteria. Patients may have met more than one exclusion criterion; therefore, exclusion counts are not additive

A total of 6,739 patients were screened for inclusion, of whom 6,051 were excluded, resulting in a final cohort of 688 patients (Fig. 1). The most common reasons for exclusion were co-diagnosis of asthma (n = 2,822), lack of eosinophil testing during the study period (n = 663), documented immunosuppression (n = 654), and insufficient follow-up data (n = 643).

**Fig. 2:**
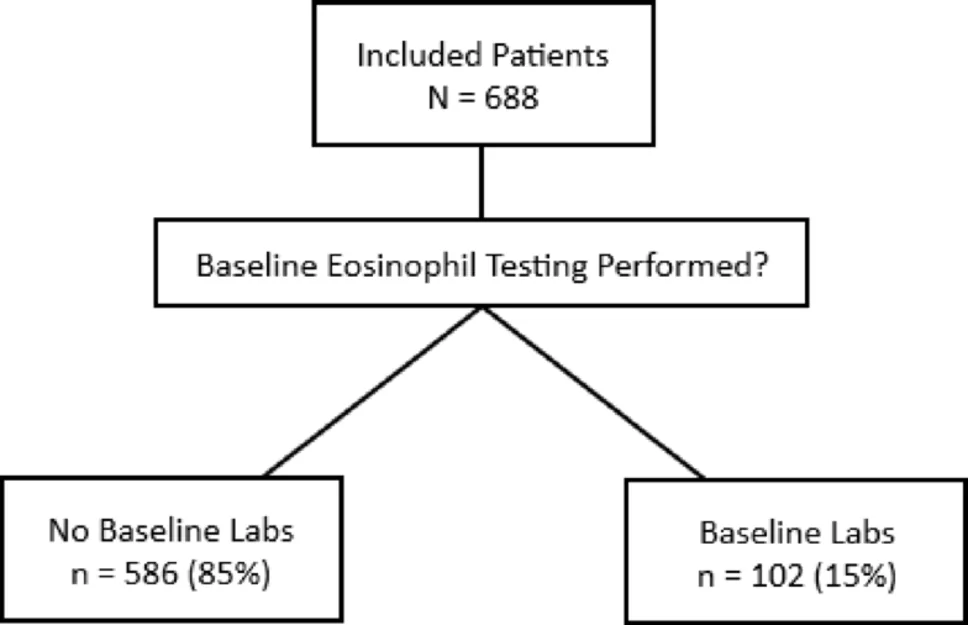
Distribution of baseline eosinophil testing among included patients. Baseline eosinophil counts were available for 102 patients (15%) and unavailable for 586 patients (85%)

The study population was evenly distributed by sex and consisted predominantly of Caucasian patients (85%), with a mean age of 69 ± 11 years. Most patients were current (37%) or former (58%) smokers. Common comorbidities included type 2 diabetes mellitus (25%) and heart failure (23%). Baseline eosinophil testing within three months prior to ICS initiation was available for 102 patients (15%) (Fig. 2).

Most patients were prescribed triple therapy consisting of ICS/LABA/LAMA (62%), while 38% received ICS/LABA therapy and only one patient received ICS/LAMA therapy. ICS-containing regimens were most initiated by pulmonologists (65%). Prescribers were primarily physicians (74%), followed by advanced practice registered nurses (21%) (Table 1).

**Table 1:** Baseline characteristics of patients initiated on inhaled corticosteroid therapy

Characteristic	N = 688
Age, years, mean ± SD	69 ± 11
Sex—no. (%)	
Male	345 (50)
Female	343 (50)
Race—no. (%)	
Black	83 (12)
White	581 (85)
Other	24 (3)
Smoking status, n (%)	
Current smoker	256 (37)
Former smoker	397 (58)
Never smoker	35 (5)
Comorbidities, n (%)	
Type 1 DM	3 (0.4)
Type 2 DM	174 (25)
Heart failure	161 (23)
Osteopenia	28 (4)
Osteoporosis	57 (8)
Bronchiectasis	22 (3)
Vaccination status, n (%)	
Influenza	354 (51)
COVID-19	455 (66)
Pneumococcal	145 (21)
Exacerbations in Prior Year	
Moderate, n (%)	59 (9)
Severe, n (%)	111 (16)
Baseline eosinophil testing occurred, n (%)	102 (15)
Baseline eosinophil count (cells/µL), median (IQR)^*^	180 (100–290)
Eosinophil category, n (%)^†^	
< 100 cells/µL	20 (20)
100–299 cells/µL	57 (56)
≥ 300 cells/µL	25 (25)
ICS Regimen, n (%)	
ICS/LAMA	1 (0.1)
ICS/LABA	259 (38)
ICS/LABA/LAMA	428 (62)
Prescriber specialty, n (%)	
Pulmonology	450 (65)
Internal medicine	110 (16)
Family medicine	53 (8)
Unknown	75 (11)
Prescriber credential, n (%)	
Doctor of Medicine (MD)	425 (69)
Doctor of Osteopathic Medicine (DO)	33 (5)
Physician assistant (PA)	25 (4)
Advanced practice registered nurse (APRN)	127 (21)
Doctor of Pharmacy (PharmD)	3 (0.5)
Unknown	75 (11)

^*^Reported among patients with available baseline eosinophil testing (n = 102)

^†^Percentages calculated among patients with available baseline eosinophil testing (n = 102)

*COPD* Chronic obstructive pulmonary disease, *ICS* Inhaled corticosteroid, *LABA* Long-acting beta agonist, *LAMA* Long-acting muscarinic antagonist, *SD* Standard deviation, *IQR* Interquartile range

### Outcomes

Most patients were prescribed ICS outside GOLD eosinophil criteria (589/688, 86%), whereas only 99 patients (14%) met guideline-based indications for ICS use. Baseline eosinophil testing was more common among patients prescribed ICS within guideline-based criteria than among those prescribed ICS outside guideline-based criteria (30% vs. 12%, *p* < 0.001). Among patients with available baseline eosinophil data, 20% had eosinophil counts < 100 cells/µL without a qualifying exacerbation history, while 42% had eosinophil counts of 100–299 cells/µL without a qualifying exacerbation history (Table 2).

**Table 2:** Outcomes by alignment of ICS prescribing with GOLD eosinophil criteria

Outcome	Total (N = 688)	Outside GOLD criteria (N = 589)	Within GOLD criteria (N = 99)	*P*-value	OR % (95% CI)
Baseline eosinophil testing, n (%)	102 (15)	72 (12)	30 (30)	< 0.001	3.1 (1.8–5.2)
Eosinophils < 100 cells/µL without exacerbation indication, n (%)^*^	20 (20)	18 (25)	2 (7)	0.034	0.2 (0.02–1.0)
Eosinophils 100–299 cells/µL without exacerbation indication, n (%)^*^	43 (42)	43 (60)	3 (10)	< 0.001	–
Eosinophils ≥ 300 cells/µL, n (%)	25 (25)	0 (0)	25 (83)		

^*^Percentages calculated among patients with baseline eosinophil testing prior to ICS initiation: total (n = 102), outside GOLD criteria (n = 72), and within GOLD criteria (n = 30). Of the 57 patients with eosinophil counts of 100–299 cells/µL, 43 did not have a qualifying exacerbation indication and are reported here

Pneumonia occurred in 102 patients (15%) during follow-up; this number coincidentally matched the number of patients with available baseline eosinophil testing, although these represent separate patient populations. The incidence of pneumonia was similar among patients prescribed ICS outside and within GOLD eosinophil criteria (15% vs. 16%, *p* = 0.686). Similar findings were observed for community-acquired pneumonia and COVID-19 pneumonia. Median time to first pneumonia was also comparable between groups (258 vs 263 days, *p* = 0.402) (Table 3).

**Table 3:** Pneumonia outcomes following initiation of inhaled corticosteroid therapy

Outcome	Total (N = 688)	Outside GOLD criteria (N = 589)	Within GOLD criteria (N = 99)	*P*-value	OR (95% CI)
Any pneumonia, n (%)^*^	102 (15)	86 (15)	16 (16)	0.686	1.1 (0.6–2.1)
Community-acquired pneumonia (CAP), n (%)	102 (15)	86 (15)	16 (16)	0.686	1.1 (0.6–2.1)
COVID-19 pneumonia, n (%)	89 (13)	76 (13)	13 (13)	0.950	1.0 (0.5–2.0)
Time to first pneumonia, median days (IQR)^†^	258 (127–493)	258 (140–501)	263 (116–409)	0.402	–

^*^The number of patients who developed pneumonia (n = 102) coincidentally matched the number of patients with available baseline eosinophil testing; these counts represent separate study measures and should not be interpreted as the same patient subgroup

^†^Reported among patients who developed pneumonia (n = 102)

ICS de-escalation was uncommon overall. De-escalation was attempted in 46 patients (7%), with successful discontinuation occurring in 31 patients (5%) and re-initiation occurring in 15 patients (2%). Median time to de-escalation was similar between patients prescribed ICS outside and within guideline-based criteria (211 vs 377 days, *p* = 0.297) (Table 4).

**Table 4:** Inhaled corticosteroid de-escalation outcomes

Outcome	Total (N = 688)	Outside GOLD criteria (N = 589)	Within GOLD criteria (N = 99)	*P*-value
Attempted de-escalation, n (%)	46 (7)	40 (7)	6 (6)	0.788
Successful discontinuation, n (%)	31 (5)	27 (5)	4 (4)	1.000
Re-initiation, n (%)	15 (2)	13 (2)	2 (2)	1.000
Time to de-escalation, median days (IQR)^*^	242 (128–444)	211 (125–444)	377 (203–451)	0.297

^*^Reported among patients who underwent attempted ICS de-escalation (n = 46)

Univariate predictors of prescribing within GOLD eosinophil criteria are presented in Table 5. A history of prior exacerbations was strongly associated with guideline-concordant prescribing, including both ≥ 2 moderate exacerbations and ≥ 1 severe exacerbation during the preceding year (*p* < 0.001 for both). Male sex was associated with lower odds of guideline-concordant prescribing (OR 0.6, 95% CI 0.4–1.0; *p* = 0.036). Most other demographic, clinical, and prescriber-related variables were not significantly associated with prescribing patterns.

**Table 5:** Univariate predictors of prescribing within GOLD eosinophil criteria

Variable	Outside GOLD criteria (N = 589)	Within GOLD criteria (N = 99)	*p*-value	OR (95% CI)
Age, years, mean ± SD	69 ± 10	69 ± 11	0.705	1.0 (1.0–1.0)
Male sex, n (%)	305 (52)	40 (40)	0.036	0.6 (0.4–1.0)
Current smoker, n (%)	217 (37)	39 (39)	0.627	1.1 (0.7–1.8)
Former smoker, n (%)	338 (57)	59 (60)	0.680	1.1 (0.7–1.7)
≥ 2 moderate exacerbations in prior year, n (%)^*^	1 (0.2)	58 (59)	< 0.001	831 (133–33,455)
≥ 1 severe exacerbation in prior year, n (%)	76 (13)	35 (35)	< 0.001	3.7 (2.2–6.1)
Triple therapy at initiation, n (%)	365 (62)	63 (64)	0.752	1.1 (0.7–1.7)
Internal Medicine vs Pulmonology	95 (16)	15 (15)	0.806	0.9 (0.5–1.7)
Family Medicine vs Pulmonology	46 (8)	7 (7)	0.799	0.9 (0.3–2.1)
DO vs MD	29 (5)	4 (4)	1.000	0.8 (0.2–2.4)
Non-physician prescriber (APRN/PA/PharmD) vs MD	103 (17)	24 (24)	0.109	1.5 (0.9–2.6)

*APRN* Advanced practice registered nurse, *CI* Confidence interval, *DO* Doctor of Osteopathic Medicine, *GOLD* Global initiative for chronic obstructive lung disease, *ICS* Inhaled corticosteroid, *MD* Doctor of Medicine, *PA* Physician assistant

^*^The odds ratio for ≥ 2 moderate exacerbations in the prior year should be interpreted cautiously because of sparse event counts

## Discussion

This retrospective cohort study evaluated ICS prescribing patterns among patients with COPD and their alignment with GOLD eosinophil criteria. Most patients were prescribed ICS outside guideline-based indications, and baseline eosinophil testing prior to treatment initiation was uncommon. Despite differences in guideline alignment, rates of pneumonia and ICS de-escalation were similar between groups. Prior exacerbation history was strongly associated with guideline-concordant prescribing, whereas most demographic and prescriber-related characteristics were not associated with prescribing patterns.

These findings are consistent with previous real-world studies demonstrating frequent ICS prescribing outside evidence-based indications in COPD management [3, 4, 11]. Observational analyses have reported that many patients receiving ICS lack a qualifying exacerbation history or elevated blood eosinophil counts, suggesting that guideline recommendations are not consistently incorporated into routine clinical practice. Similarly, only a minority of patients in the present study underwent baseline eosinophil testing prior to ICS initiation, indicating limited use of eosinophil-guided decision-making.

Since 2019, GOLD guidelines have recommended the use of blood eosinophil counts to guide ICS therapy, with higher eosinophil counts associated with greater reductions in exacerbation risk [1, 2, 5, 6]. Despite these recommendations, implementation remains variable. Among patients with available baseline eosinophil testing, a substantial proportion did not meet guideline-based indications for ICS use, further highlighting the gap between guideline recommendations and clinical practice.

Clinical outcomes were similar regardless of guideline alignment. Although previous studies have reported an increased risk of pneumonia associated with ICS use, particularly among patients without evidence-based indications for therapy [11, 12], no significant differences in pneumonia incidence were observed in the present study. Similarly, ICS de-escalation was infrequent, suggesting that ICS therapy may not be routinely reassessed after initiation, even among patients who may not meet criteria for continued treatment.

Several factors may explain the lower-than-expected pneumonia incidence observed in this study. Pneumonia events occurring outside the Cleveland Clinic health system may not have been captured, potentially resulting in underestimation of event rates. Additionally, the study period overlapped with the COVID-19 pandemic, during which public health measures may have reduced exposure to respiratory pathogens and lowered rates of respiratory infections [16].

This study has several inherent limitations. First, the retrospective design relied on the accuracy and completeness of electronic medical record documentation. COPD diagnoses were identified using ICD-10 codes and were not confirmed through review of pulmonary function testing, introducing the potential for misclassification. Second, eosinophil testing performed outside the Cleveland Clinic health system may not have been captured, and eosinophil values may have been affected by recent corticosteroid exposure despite efforts to minimize this limitation through study design. Third, medication adherence could not be confirmed, and prescriptions obtained outside the health system may not have been identified. Finally, the low frequency of some outcomes, particularly ICS de-escalation, may have limited the ability to detect differences between groups.

Overall, these findings demonstrate that ICS prescribing frequently occurs outside GOLD eosinophil criteria and that baseline eosinophil testing remains underutilized in routine practice. Greater integration of eosinophil-guided decision-making may improve alignment with guideline-directed COPD management and identify opportunities for more appropriate use of ICS therapy.

## Conclusions

Current GOLD guidelines recommend inhaled corticosteroid (ICS) use in select patients with chronic obstructive pulmonary disease (COPD) based on exacerbation history and blood eosinophil counts. In this retrospective cohort study, most patients were prescribed ICS outside of GOLD eosinophil criteria, and baseline eosinophil testing was infrequently performed prior to treatment initiation. These findings are consistent with previous real-world studies demonstrating limited implementation of eosinophil-guided prescribing in clinical practice.

Despite substantial differences in guideline alignment, clinical outcomes including pneumonia incidence and rates of ICS de-escalation were similar between patients prescribed ICS within and outside GOLD eosinophil criteria. Prior exacerbation history was strongly associated with guideline-concordant prescribing, while most demographic and prescriber-related factors were not associated with prescribing patterns.

These findings highlight opportunities to improve the integration of eosinophil testing into routine COPD management and promote greater adherence to guideline-directed ICS prescribing. Future studies should evaluate interventions aimed at increasing eosinophil-guided decision-making, identify opportunities for appropriate ICS de-escalation, and determine the impact of these strategies on prescribing patterns and patient outcomes.
